# Deoxyschizandrin, Isolated from Schisandra Berries, Induces Cell Cycle Arrest in Ovarian Cancer Cells and Inhibits the Protumoural Activation of Tumour-Associated Macrophages

**DOI:** 10.3390/nu10010091

**Published:** 2018-01-15

**Authors:** Kijun Lee, Ji-Hye Ahn, Kyung-Tae Lee, Dae Sik Jang, Jung-Hye Choi

**Affiliations:** 1 Department of Life and Nanopharamceutical Sciences, Kyung Hee University, Seoul 02447, Korea; standardlee7@gmail.com (K.L.); jihyeahn21@gmail.com (J.-H.A.); ktlee@khu.ac.kr (K.-T.L.); dsjang@khu.ac.kr (D.S.J.); 2 College of Pharmacy, Kyung Hee University, Seoul 02447, Korea

**Keywords:** deoxyschizandrin, Schisandra berries, ovarian cancer, cell cycle arrest, tumour-associated macrophage

## Abstract

Deoxyschizandrin, a major lignan of Schisandra berries, has been demonstrated to have various biological activities such as antioxidant, hepatoprotective, and antidiabetic effects. However, the anti-cancer effects of deoxyschizandrin are poorly characterized. In the present study, we investigated the anti-cancer effect of deoxyschizandrin on human ovarian cancer cell lines and tumour-associated macrophages (TAMs). Deoxyschizandrin induced G_0_/G_1_ phase cell cycle arrest and inhibited cyclin E expression in human ovarian cancer cells. Overexpression of cyclin E significantly reversed the deoxyschizandrin-induced cell growth inhibition. Interestingly, increased production of reactive oxygen species and decreased activation of Akt were observed in A2780 cells treated with deoxyschizandrin, and the antioxidant compromised the deoxyschizandrin-induced cell growth inhibition and Akt inactivation. Moreover, deoxyschizandrin-induced cell growth inhibition was markedly suppressed by Akt overexpression. In addition, deoxyschizandrin was found to inhibit the expression of the M2 phenotype markers CD163 and CD209 in TAMs, macrophages stimulated by the ovarian cancer cells. Moreover, expression and production of the tumour-promoting factors MMP-9, RANTES, and VEGF, which are highly enhanced in TAMs, was significantly suppressed by deoxyschizandrin treatment. Taken together, these data suggest that deoxyschizandrin exerts anti-cancer effects by inducing G_0_/G_1_ cell cycle arrest in ovarian cancer cells and reducing the protumoural phenotype of TAMs.

## 1. Introduction

The fruits of *Schisandra chinensis* (Schisandra berries), also known as five-flavour-fruit, are widely used in East Asia as a food substance and medicinal herb. In Korea and China, it is called omiza and wu-wei-zi, respectively, and is commonly used in teas, jam, wine, and many other products as a nutritional supplement. In traditional medicine, Schisandra berries are used to treat various symptoms such as cough, fatigue, spontaneous sweating, dysentery, and insomnia [1,2]. A phytochemical study revealed that Schisandra berries contain many dibenzocyclooctadiene derivatives in different amounts [2]. Modern pharmacological studies have shown that deoxyschizandrin, a major dibenzocyclooctadiene lignan present in Schisandra berries [3], possesses a wide range of bioactivities, including neuroprotective [4], hepatoprotective [5], antioxidant [6], antiviral [7], and antidiabetic effects [8]. However, the anti-cancer effects of deoxyschizandrin are poorly characterized. In this study, we aimed to elucidate the inhibitory effect of deoxyschizandrin on growth of human ovarian cancer cells and protumoural activation of tumour-associated macrophages (TAMs).

Cell cycle is a complex process involved in the growth and proliferation of cells. Abnormalities in the expression of cell cycle regulatory genes resulting in elevated proliferative capacity have been observed in almost all human cancers [9]. Cell growth progresses in systematic steps through G_1_, S, G_2_, and M phases of the cell cycle and is controlled by the interdependent activity of cell cycle regulatory proteins [10]. These regulatory proteins are cyclin dependent kinases (CDKs) and the proteins that modulate their activity, cyclins and cyclin-dependent kinase inhibitors (CKIs) [11]. Blockage of the cell cycle by regulating those proteins has been regarded as an effective strategy for the suppression of uncontrolled growth of cancer cells [12].

It has been suggested that circulating macrophages accumulate in tumours and change their microenvironment to accelerate tumour progression [13]. Macrophages have been shown to alter their functional phenotypes in response to diverse signals generated from tumour and non-tumour cells. Recent studies have demonstrated that TAMs are key factor in tumour microenvironment and closely resemble the M2-phenotype macrophages which possess various protumoural properties [14]. For example, TAMs have been shown to stimulate cancer metastasis, angiogenesis, immune suppression, and chemoresistance [15]. In addition, TAM infiltration has been associated with poor clinical outcomes [16]. In this regard, TAMs are considered as a potential therapeutic target for cancer treatment.

## 2. Materials and Methods

### 2.1. Sample Preparation

Deoxyschizandrin and schizandrin used for the present study were prepared in our previous study [17]. Briefly, the dried fruits of *Schisandra chinensis* Baillon (3.5 kg) were extracted with 10 L of 80% aqueous EtOH three times by maceration. The extracts were concentrated in vacuo at 40 °C to give an 80% EtOH extract (1.5 kg). The 80% EtOH extract (1.5 kg) was suspended in distilled water (5 L) and then partitioned with n-hexane, EtOAc, and BuOH, successively. A portion of the *n*-hexane-soluble layer (125 g) was subjected to silica gel (70–230 mesh) column chromatography (CC) and eluted with *n*-hexane-EtOAc (1:0 to 0:1, *v*/*v*) to afford 7 fractions (H1–H7). Fraction H3 (17.1 g) was subjected to a column chromatography on silica gel (7.9 × 46 cm, 70–230 mesh) eluted with *n*-hexane-EtOAc (9:1 to 7:3, *v*/*v*) to afford 7 subfractions (H3-1-H3-10). Deoxyschizandrin (826.4 mg) was obtained by purifying fraction H3-4 (2.6 g) using Sephadex LH-20 CC (3.6 × 72 cm, CH_2_Cl_2_-MeOH, 1:1 *v*/*v*). The EtOAc-soluble layer (80 g) was chromatographed over silica gel CC as stationary phase with a CH_2_Cl_2_-acetone mixture (49:1 to 0:1, *v*/*v*) as mobile phase to afford 11 pooled fractions (E1-E11). Fraction E5 (4.17 g) was successively fractionated using a Sephadex LH-20 with CH_2_Cl_2_-MeOH mixture (1:1, *v*/*v*) and silica gel CC (3.9 × 24.5 cm, *n*-hexane-EtOAc, 7:3 *v*/*v*) to yield schizandrin (330 mg). The purity of these compounds (>95%) was determined by HPLC and NMR.

### 2.2. Materials

Roswell Park Memorial Institute (RPMI) 1640, fetal bovine serum (FBS), penicillin, and streptomycin were obtained from Life Technologies Inc. (Grand Island, NY, USA). 3-(4,5-Dimethylthiazol-2-yl)-2,5-diphenyl-tetrazolium bromide (MTT) was purchased from Molecular Probes Inc. (Eugene, OR, USA). Propidium iodide (PI), *N*-acetyl-l-cystein (NAC), 2-mercaptoethanol, and phorbol myristate acetate (PMA) were obtained from Sigma Chemical (St. Louis, MO, USA). Phenylmethylsulfonylfluoride (PMSF) was purchased from BD Biosciences (San Jose, CA, USA). Antibodies for total Akt, cyclin E, MMP-9, β-actin, and dichlorofluorescein diacetate (DCFH-DA) were purchased from Santa Cruz Biotecnology (Santa Cruz, CA, USA). Phospho-Akt antibody was obtained from Cell Signaling (Beverly, MA, USA).

### 2.3. Cell Culture

Three human ovarian cancer cell lines (A2780, SKOV3, and OVCAR3) and human monocytic cell line (THP-1) were originally from American Type Culture Collection (Rockville, MD, USA). A2780 and SKOV3 cells were cultured in RPMI 1640 supplemented with 5% fetal bovine serum (FBS), penicillin (100 U/mL), and streptomycin sulfate (100 µg/mL). OVCAR3 cells were cultured in RPMI 1640 supplemented with 10% FBS, penicillin (100 U/mL), and streptomycin sulfate (100 µg/mL). THP-1 cells were cultured in RPMI 1640 supplemented 5% FBS, penicillin (100 U/mL), streptomycin sulfate (100 µg/mL), and 0.05 mM of 2-mercaptoethanol. THP-1 cells are differentiated into macrophage with 100 nM of phorbol myristate acetate (PMA) for 24 h. Tumour-associated macrophages (TAMs) were prepared by stimulating THP-1 monocytic cells with conditioned medium from A2780 ovarian cancer cells. Conditioned medium of A2780 cells were obtained after 24 h of cell seeding.

### 2.4. MTT Assay

Cell viability was determined using MTT assay. First, the cells were seeded in each well containing 50 µL of RPMI medium in 96-well plates. After 24 h, various concentrations of deoxyschizandrin were added. After 48 h, 25 µL of MTT (5 mg/mL stock solution) was added, and then the plates were incubated for an additional 4 h. The medium was discarded and the formazan crystals that formed in the cells were dissolved in 50 µL of dimethyl sulfoxide (DMSO). The optical density was measured at 540 nm using a SpectraMax (Molecular Devices, Sunnyvale, CA, USA). Each assay was performed in duplicate or triplicate.

### 2.5. Propidium Iodide (PI) Staining for Cell Cycle Analysis

At the time of collection, the cells were harvested and washed twice with ice-cold PBS. The cells were fixed and permeabilized with 70% ice-cold ethanol at −20 °C for 4 h. The cells were washed once with PBS and resuspended in a staining solution containing propidium iodide (5 mg/mL) and RNase A (1 µg/mL). The cell suspensions were incubated for 20 min at room temperature. Then, the cell cycle profiles were measured by Guava EasyCyte Mini (Millipore, Billerica, MA, USA) detecting 10,000 cells per each group.

### 2.6. Western Blot Analysis

Cells were washed with ice-cold PBS and extracted in protein lysis buffer (Intron Biotechnology, Seoul, Korea). The total protein content of the samples was determined according to the Bradford assay (Bio-Rad Laboratories, Hercules, CA, USA) using a bovine serum albumin (BSA) standard curve. Protein samples of cell lysate were mixed within equal volume of SDS sample buffer, boiled for 5 min and were loaded on 10–12% SDS-PAGE gels. After acrylamide gel-electrophoresis, proteins were transferred to polyvinylidene difluoride (PVDF) membranes. The membranes were blocked in 5% non-fat dry milk for 30 min, washed and were incubated for overnight at 4 °C with specific primary antibodies against cyclin E, total Akt, phospho-Akt, and β-actin in Tris-buffered saline (TBS) containing Tween-20 (0.1%). Primary antibodies were removed by washing the membranes three times in TBS-T and the membranes were incubated for 2 h with horseradish peroxidase-conjugated secondary antibody (1:1000–2000). Following the washing procedures in TBS-T, immune-positive bands were visualized by enhanced chemiluminescene and exposed to ImageQuant LAS-4000 (Fujifilm Life Science, Tokyo, Japan). The relative intensities of protein bands were determined by NIH Image 1.59 software (Image J) (NIH, Bethesda, MD, USA) and then all values were subsequently normalized to untreated group.

### 2.7. Measurement of Reactive Oxygen Species (ROS)

The intracellular accumulation of ROS was examined using the fluorescent probe DCFH-DA. Cells were harvested and suspended in PBS. After being pretreated with deoxyschizandrin for 30 min, DCFH-DA (20 mM) were added and incubated for 30 min at 37 °C. Levels of DCF formation were measured by Guava EasyCyte Mini.

### 2.8. RNA Extraction and Real-Time RT-PCR Analysis

Total cellular RNA was extracted using Easy Blue^®^ kits (Intron Biotechnology, Seoul, Korea) according to the manufacturer’s instruction. Total RNA (500 ng) was reverse transcribed into first strand cDNA (Amersham Pharmacia Biotech, Oakville, ON, Canada) following the manufacturer’s protocol. The synthesized cDNA was used as a template for polymerase chain reaction (PCR) amplification. Real-time PCR was performed using a SYBR Premix Ex Taq™ Kit (TaKaRa, Kyoto, Japan) and Thermal Cycler Dice RealTime PCR System (TaKaRa). Searching and selection of primer sequences were performed using the Primer-BLAST tool available at the National Center for Biotechnology Information (NCBI) website and the Primer3Plus online tool. The primers used for real-time RT-PCR were as follows: for CD163 sense primer, 5′-AGC AGG GAT GTT GGA GTA GT-3′, and anti-sense primer, 5′-TAA GCT GCT GGC AAA GAA CA-3′, for CD209 sense primer, 5′-CCA GGA TGG TCT CGA TCT CT-3′, for anti-sense primer, 5′-CAG CGA GGA AGA AAC CTA CC-3′, for MMP-9 sense primer, 5′-GGA CGA TGC CTG CAA CGT-3′ and anti-sense primer, 5′-CAA ATA CAG CTG GTT CCC AAT CT-3′, for RANTES sense primer, 5′-GGG TTC GGG AGT ACA TCA AC-3′ and anti-sense primer, 5′-CTG TGT GGT AGA ATC TGG GC-3′, for VEGF sense primer, 5′-ATG GCA GAA GGA GGA GGG CA-3′, and anti-sense primer, 5′-ATC GCA TCA GGG GCA CAC AG-3′, for β-actin sense primer, 5′-CAA ACA TGA TCT GGG TCA TC-3′, for anti-sense primer, 5′-GCT CGT CGT CGA CAA CGG CT-3′. A dissociation curve analysis displayed a single peak. PCRs were performed for 45–50 cycles using the following condition: denaturation at 95 °C for 5 s, annealing at 57 or 59 °C for 10 s, and elongation at 72 °C for 20 s. Mean cycle threshold (Ct) of the gene of interest was calculated from duplicate measurements and normalized with the mean Ct of a control gene, β-actin.

### 2.9. Enzyme-Linked Immunosorbent (ELISA) Assay

ELISA kits for RANTES and VEGF were purchased from Koma Biotech, Inc. (Seoul, South Korea). Cell culture media were used to measure the production levels of RANTES and VEGF by following the manufacturer’s instruction. All values were subsequently normalized to control group.

### 2.10. Transfection of Expression Vector

RC/CMV cyclin E (plasmid #8963), 1036 pcDNA3 Myr HA Akt1 (plasmid #9008), and pcDNA3.1 empty vector were purchased from Addgene (Cambridge, MA, USA). A2780 cells were transfected with the indicated vectors using Lipofectamine™ LTX transfection reagent (Invitrogen, Carlsbad, CA, USA) according to the manufacturer’s protocol. The data of validation using Western blotting are shown in Figure S1.

### 2.11. Statistical Analysis

The data are prepared as the mean ± SD. One-way analysis of variance (ANOVA) was used to identify statistically significant differences. We used GraphPad Prism software for statistical analyses and graphs (GraphPad, San Diego, CA, USA). *p*-values < 0.05 were considered to be statistically significant.

## 3. Results

### 3.1. Deoxyschizandrin Induces G_0_/G_1_ Cell Cycle Arrest in Human Ovarian Cancer Cells

**Figure 1:**
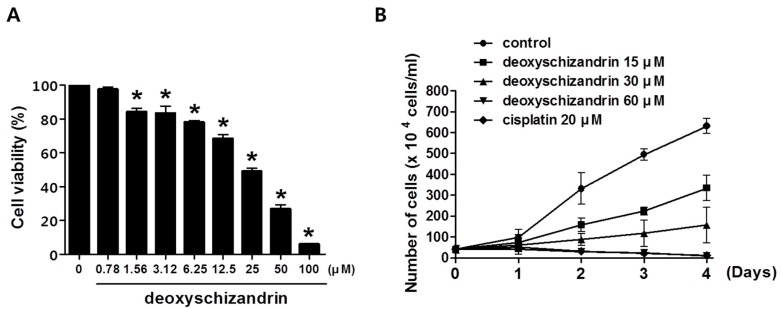
Effect of deoxyschizandrin on cell growth in human ovarian cancer cells: (**A**) Effect of deoxyschizandrin on cell viability in A2780 cells was measured using MTT assay. A2780 cells were seeded at concentration of 1.0 × 10^5^ cells/well in a 96-well plate. Then, the cells were treated with the indicated concentration of deoxyschizandrin for 48 h. The values represent the mean ± SD of results from three independent experiments. * *p* < 0.05 vs. the control group; (**B**) Effect of deoxyschizandrin on cell growth in A2780 cells was determined by cell counting. Growing cells were treated with the indicated concentration of deoxyschizandrin and cisplatin for 1–4 days (● control, ■ 15 µM, ▲ 30 µM, ▼ 60 µM, ♦ cisplatin 20 µM). Cisplatin was used as a positive control. The data shown represent the mean ± SD of at least two independent experiments.

The effect of deoxyschizandrin on the viability of human ovarian cancer cells was first investigated using MTT assay. Deoxyschizandrin showed a substantial growth inhibitory effect in human ovarian cancer cell lines A2780, OVCAR3, and SKOV3 with IC_50_ values of 27.81, 70.34, and 67.99 µM respectively (Table 1). It is of note that the IC_50_ value of schizandrin, which differs from deoxyschizandrin by a single hydroxyl group present on the cyclooctene ring, was over 100 µM for all three ovarian cancer cell lines. We further investigated the effect of deoxyschizandrin at various concentrations on the growth of A2780 cells at time intervals of 24, 48, 72, and 96 h (Figure 1). Deoxyschizandrin showed cytostatic activity at concentrations ranging from 15 to 30 µM in A2780 cells.

**Figure 2:**
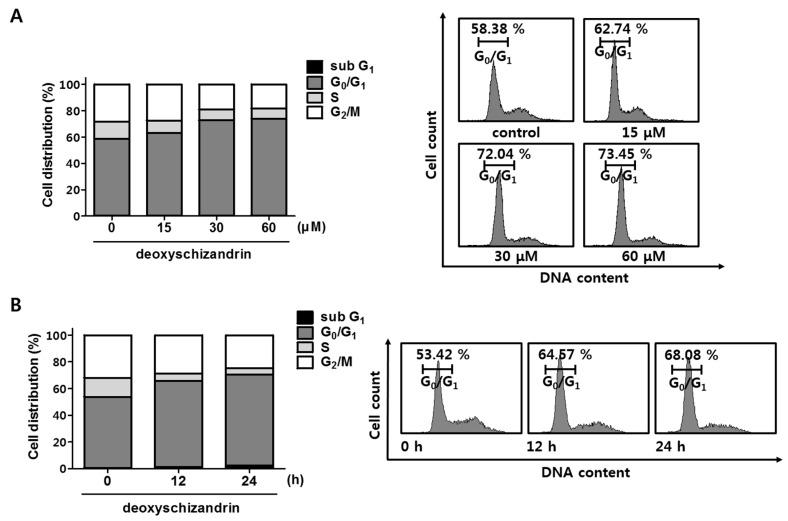
Effect of deoxyschizandrin on cell cycle in A2780 cells: (**A**) Cell cycle analysis was performed using propidium iodide (PI) staining assay. A2780 cells were treated with the indicated concentration of deoxyschizandrin (15, 30, and 60 µM) for 48 h and then stained with propidium iodide (PI). The cell cycle distribution profiles of the cells were determined by flow cytometry (FACS); (**B**) Distribution of cell number in cell cycle was measured at 12 and 24 h after deoxyschizandrin (30 µM) treatment.

**Figure 3:**
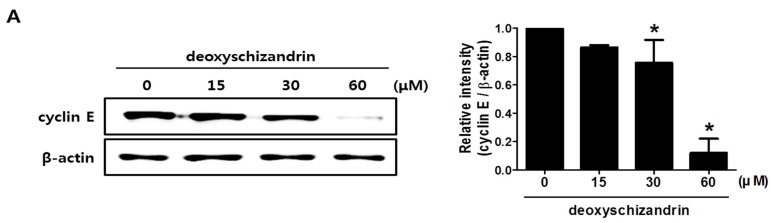

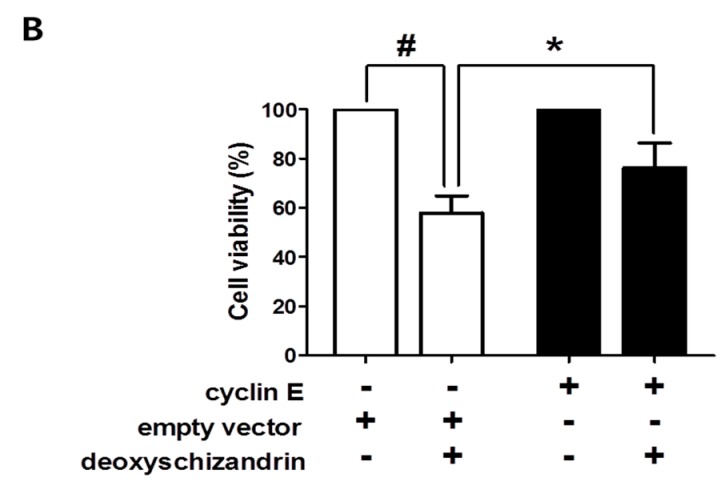
Involvement of cyclin E in deoxyschizandrin-induced cell cycle arrest in A2780 cells: (**A**) Effect of deoxyschizandrin on cyclin E expression was measured using Western blotting. A2780 cells were treated with the indicated concentration of deoxyschizandrin (15, 30, and 60 µM) for 48 h. β-Actin was used as an internal control. Band images are representatives of three individual experiments and the results of densitometric analysis represent the mean ± SD of three independent experiments. * *p* < 0.05 vs. the control group; (**B**) Involvement of cyclin E in deoxyschizandrin-induced cell cycle arrest was examined using MTT assay. A2780 cells were transfected with cyclin E expression vector and were treated with deoxyschizandrin (30 µM) for 48 h. The values represent the mean ± SD of results from three independent experiments. # *p* < 0.05 vs. the control group; * *p* < 0.05 vs. the treated group transfected with empty vector.

Next, cell cycle analysis was performed to determine whether the growth inhibitory effect of deoxyschizandrin is associated with cell cycle arrest. As shown in Figure 2, deoxyschizandrin treatment significantly increased the number of cells in the G_0_/G_1_ phase in a dose- and time-dependent manner while the number of apoptotic and necrotic cells (sub G_1_ phase) remained unchanged. In addition, the expression of cyclin E, a key regulatory protein for G_1_ phase, was markedly inhibited (Figure 3A), and cyclin E overexpression significantly compromised the deoxyschizandrin-induced cell growth inhibition in A2780 cells (Figure 3B). These findings indicate that deoxyschizandrin inhibits ovarian cancer cell growth through induction of G_0_/G_1_ cell cycle arrest.

### 3.2. ROS Production Is Involved in the Deoxyschizandrin-Induced Cell Growth Inhibition

**Figure 4:**
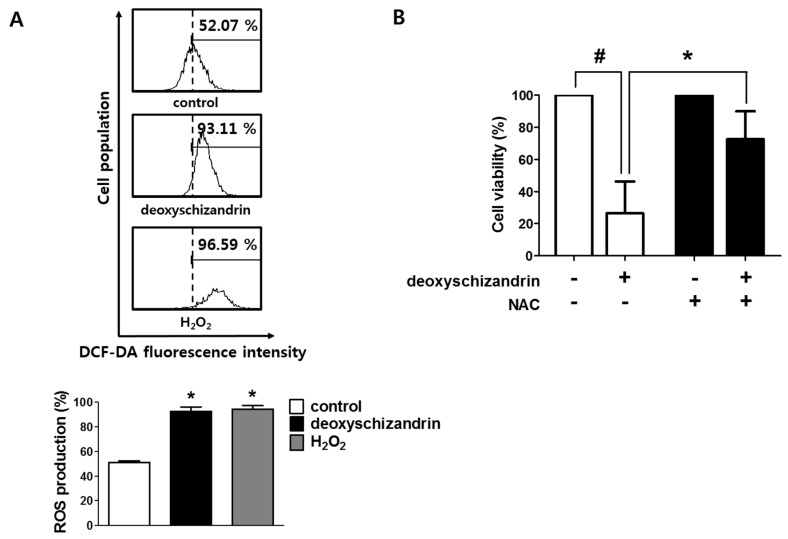
Involvement of intracellular ROS in deoxyschizandrin-induced cell cycle arrest in A2780 cells: (**A**) Generation of intracellular ROS was measured using DCFH-DA assay after deoxyschizandrin (30 µM) treatment for 30 min. The values represent the mean ± SD of results from three independent experiments. * *p* < 0.05 vs. the control group; (**B**) Involvement of intracellular ROS in deoxyschizandrin-induced cell cycle arrest in A2780 cells was examined using MTT assay. A2780 cells were treated with 5 mM of *N*-acetyl-l-cystein (NAC) and deoxyschizandrin (30 µM) for 48 h. NAC was used for ROS inhibition. The values represent the mean ± SD of results from three independent experiments. # *p* < 0.05 vs. the control group; * *p* < 0.05 vs. the treated group.

A disproportional increase in intracellular reactive oxygen species (ROS) can induce cancer cell cycle arrest, apoptosis, and cellular senescence [18]. Thus, we determined the effect of deoxyschizandrin on intracellular ROS levels in A2780 cells. As shown in Figure 4A, deoxyschizandrin considerably induced ROS production in A2780 cells. To further confirm the involvement of ROS in deoxyschizandrin-induced cell growth inhibition, an antioxidant *N*-acetyl-l-cystein (NAC) was used. NAC treatment significantly reversed deoxyschizandrin-induced decrease in A2780 cell growth (Figure 4B).

### 3.3. The Akt Pathway Is Involved in Deoxyschizandrin-Induced Cell Growth Inhibition

**Figure 5:**
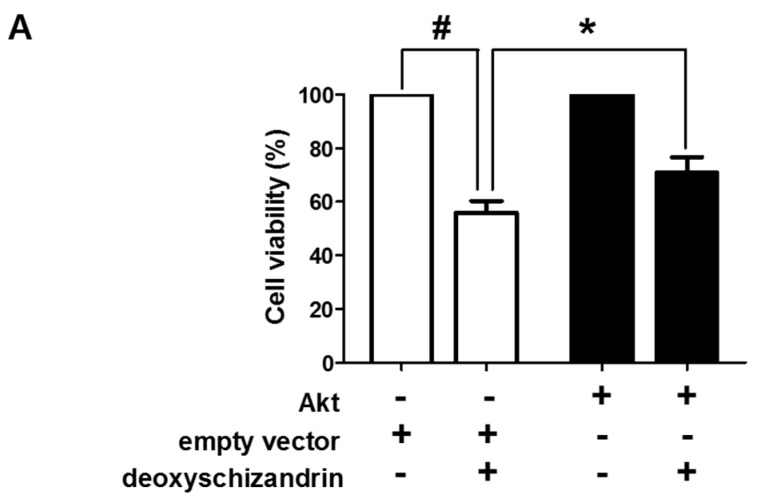

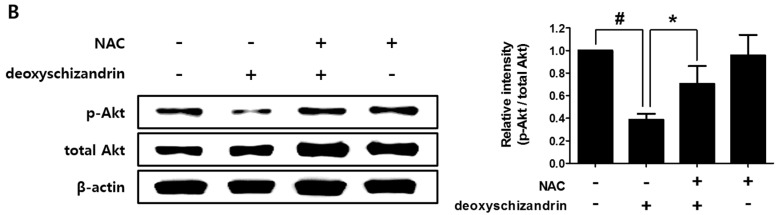
Involvement of Akt signalling in deoxyschizandrin-induced cell cycle arrest in A2780 cells: (**A**) A2780 cells were transfected with Akt expression vector and were treated with deoxyschizandrin (30 µM) for 48 h. MTT assay was performed to measure the cell viability in A2780 cells. The values represent the mean ± SD of results from three independent experiments. # *p* < 0.05 vs. the control group; * *p* < 0.05 vs. the treated group transfected with empty vector; (**B**) A2780 cells were treated with 5 mM of *N*-acetyl-l-cystein (NAC) and deoxyschizandrin (30 µM) for 8 h. Western blot analysis was performed to measure the phosphorylation of Akt in A2780 cells. β-Actin was used as an internal control. Band images are representatives of three individual experiments and the results of densitometry represent the mean ± SD of three independent experiments. # *p* < 0.05 vs. the control group; * *p* < 0.05 vs. the treated group.

Akt is a critical kinase protein that regulates various biological processes, including cell survival and proliferation. ROS generation is related with the PI3K/Akt signalling pathway [19]. First, the involvement of the PI3K/Akt pathway in the deoxyschizandrin-induced growth inhibition was investigated. Overexpression of the constitutive form of Akt significantly reversed the deoxyschizandrin-induced growth inhibition (Figure 5A). We confirmed the effect of deoxyschizandrin on Akt activation and the involvement of ROS. Deoxyschizandrin markedly suppressed the levels of Akt phosphorylation in A2780 cells and Akt dephosphorylation was significantly blocked in the presence of NAC (Figure 5B). These results suggest that the deoxyschizandrin-induced ROS production affects the PI3K/Akt pathway, resulting in the growth inhibition of ovarian cancer cells.

### 3.4. Deoxyschizandrin Inhibited the Expression of M2 Phenotype Markers in Macrophages Stimulated by Ovarian Cancer Cells

**Figure 6:**
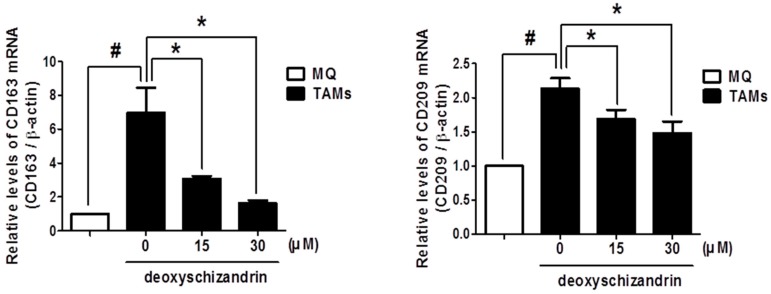
Effect of deoxyschizandrin on the expression of CD163 and CD209 in tumour-associated macrophages (TAMs); THP-1 cells were stimulated with conditioned medium from A2780 cells and were treated with the indicated concentration of deoxyschizandrin (15 and 30 µM) for 48 h. Real-time RT-PCR was conducted to measure the mRNA levels of CD163 and CD209 in macrophages. The values represent the mean ± SD of results from three independent experiments. # *p* < 0.05 vs. MQ; * *p* < 0.05 vs. the untreated TAMs.

Emerging evidence demonstrates that TAMs play an important role in cancer progression. Interestingly, TAMs have been shown to display typical M2 macrophage phenotypes [20]. We examined the effect of deoxyschizandrin on the expression of the M2 phenotype markers CD163 and CD209 in TAMs [14,21]. TAMs, macrophages stimulated by the conditioned medium of A2780 cells, were found to have higher levels of M2 phenotype markers CD163 and CD209 than control macrophages (MQ), and deoxyschizandrin significantly suppressed CD163 and CD209 expression in TAMs (Figure 6).

### 3.5. Deoxyschizandrin Inhibited the Expression of Cancer-Promoting Factors in Macrophages Stimulated by Ovarian Cancer Cells

**Figure 7:**
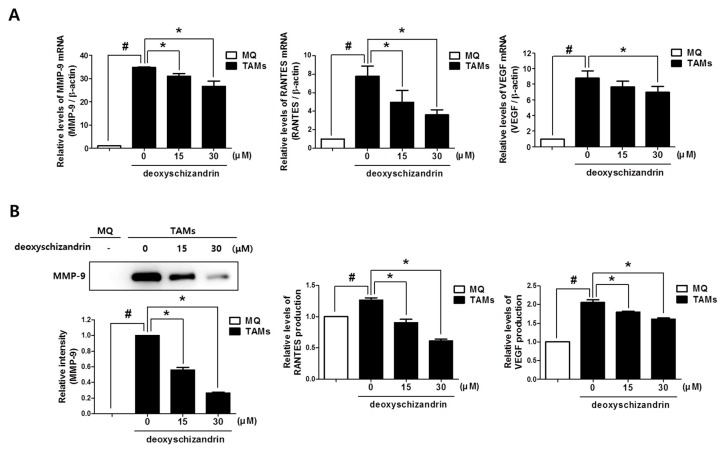
Effect of deoxyschizandrin on the mRNA expression and production of MMP-9, RANTES, and VEGF in tumour-associated macrophages (TAMs); THP-1 cells were stimulated with conditioned medium from A2780 cells and were treated with the indicated concentration of deoxyschizandrin (15 and 30 µM) for 48 h. (**A**) Real-time RT-PCR was conducted to measure the mRNA levels of MMP-9, RANTES, and VEGF in macrophages; (**B**) Western blot analysis was performed to measure the production levels of MMP-9 in cell culture media. Band images are representatives of three individual experiments and the results of densitometry represent the mean ± SD of three independent experiments. Production levels of RANTES and VEGF in cell culture media were measured using ELISA assay. The values represent the mean ± SD of results from three independent experiments. # *p* < 0.05 vs. MQ; * *p* < 0.05 vs. the untreated TAMs.

Previous research has demonstrated that TAMs can promote tumour growth, metastasis, and angiogenesis by secreting of various mediators [13]. We established that the protumoural factors MMP-9, RANTES, and VEGF are highly expressed in TAMs, compared to their expression in control macrophages (MQ) (Figure 7). Deoxyschizandrin treatment significantly suppressed the mRNA and production levels of MMP-9, RANTES, and VEGF expression in TAMs. These data suggest that deoxyschizandrin can reduce the pro-tumour activities of TAMs in tumour microenvironment by suppressing pro-tumour mediator production as well as M2 polarization.

## 4. Discussion

Ovarian cancer has the lowest five-year survival rate among gynaecologic cancers. Globally, approximately 240,000 women are diagnosed with ovarian cancer each year [22]. In 2017, approximately 22,000 new cases and 15,000 deaths were estimated in the United States alone [23]. Early detection of ovarian cancer is often missed because of its unclear symptoms while medicinal treatment at late stages is generally unsuccessful [24]. Treatments such as chemotherapy can put patients under a lot of physical stress and further deteriorate their condition [25]. Therefore, there is increasing interest in novel and alternative therapeutics to treat ovarian cancer. Many natural compounds derived from plants have long been considered as potential drug candidates for cancer especially due to their relatively toxicity and good efficacy in comparison to synthetic agents [26].

Lignans, characteristic and major components of Schisandraceae plants, are commonly classified into the following types: dibenzocyclooctadiene lignans (Type A), spirobenzofuranoid dibenzocyclooctadiene lignans (Type B), 4-aryltetralin lignans (Type C), 2,3-dimethyl-1,4-diarylbutane lignans (Type D), and 2,5-diaryltetrahydrofuran lignans (Type E) [3]. Many dibenzocyclooctadiene lignans have been isolated from Schisandra berries and their potential biological activities have been demonstrated. For example, their antioxidant [27] and hepatoprotective [28] activities have been well characterized. As for anti-cancer activity, schizandrin C and gomisin N have been reported to possess significant anti-tumoural activities by inhibiting human herpesvirus early antigen activation [29] and enhancing doxorubicin-induced apoptosis in human hepatic cancer cells [30], respectively. Deoxyschizandrin, a major dibenzocyclooctadiene lignan in Schisandra berries, has also been suggested to enhance the responsiveness of lung cancer cells to anti-cancer drugs [30,31]. However, the direct effects of deoxyschizandrin alone on the growth of cancer cells are poorly characterized. In this study, we demonstrated that deoxyschizandrin, but not schizandrin with a hydroxyl group in a saturated ring structure, markedly inhibits the viability of human ovarian cancer cells. We can reasonably speculate that the hydroxyl group is responsible for the different effects of deoxyschizandrin and schizandrin on cell viability. The hydroxyl group may allow for differential binding of the compound to target molecules. The detailed molecular mechanisms underlying the structure-activity relationship require further elucidation.

The mammalian cell cycle is a highly organized and regulated process that ensures duplication of genetic material and cell division. Proliferation of cells is regulated by several cyclin dependent kinases (CDKs) that act in complex with their cyclin subunits through distinct phases of cell cycle (G_0_/G_1_, S, G_2_, and M). Aberrant activation of these kinases and their cyclin subunits, which is frequently seen in human cancers, has provided a promising target for discovering inhibitors of cyclin or CDKs as anti-cancer drugs [32]. Cyclin E, a regulatory subunit of cyclin dependent kinase-2, is thought to be a rate limiting factor for the G_1_/S transition during the mammalian cell cycle [33]. In addition, Marone et al. have suggested that cyclin E is a key regulatory protein in ovarian cancer progression [34]. In this study, we demonstrated that deoxyschizandrin suppresses cyclin E expression and induces G_0_/G_1_ cell cycle arrest in human ovarian cancer cells. Additionally, we confirmed that cyclin E downregulation was, at least partially, required for deoxyschizandrin-induced cell growth inhibition.

ROS play a critical role in various human diseases including cancer [35]. Under normal physiological conditions, cells control ROS levels by adjusting cell signalling and homeostasis. However, in oxidative stress conditions, excessive ROS can produce harmful effects by damaging cellular proteins, lipids, and DNA [36]. In addition, numerous studies have demonstrated that disproportional increases in ROS can induce apoptotic cell death and growth arrest in various cancer cell types [18]. Many chemotherapeutic agents reportedly exhibit anti-tumour properties by enhancing the intracellular ROS level [37]. In addition to synthetic agents, several natural compounds show anti-cancer activity by regulating ROS in cancer cells. For example, Chen et al. have demonstrated that bisdemethoxycurcumin isolated from *Curcuma longa*, increases intracellular ROS levels, resulting in cell cycle arrest in human liver cancer [38]. Dibenzocyclooctadiene lignans including deoxyschizandrin have been reported to possess high antioxidant potentials [6,39]. However, we discovered that deoxyschizandrin induced ROS production to inhibit the growth of human ovarian cancer cells. In fact, many antioxidant natural compounds have been demonstrated to similarly act as a pro-oxidant in some human cancer cells [40]. The distinctive molecular mechanisms by which those compounds including deoxyschizandrin induce ROS production in human cancer cells should be further investigated. It is worth considering that ROS production was responsible for the inhibition of the PI3K/Akt pathway by deoxyschizandrin in human ovarian cancer cells. Jin et al. have suggested that ROS generation is closely related with the Akt signalling pathway [41]. The major functions of Akt are promotion of growth factor-mediated cell proliferation through phosphorylation of many physiological substrates and inhibition of apoptosis through the inactivation of pro-apoptotic proteins [42]. The Akt signalling pathway is usually impaired in several cancers and is associated with tumour malignancy. Thus, this signalling pathway has been regarded as a promising target for cancer therapy and cancer prevention [43].

Macrophages alter their functional phenotypes in response to various signals generated from tumour and stromal cell microenvironments [20]. According to their functions, macrophages are divided broadly into two phenotypes, classical M1 and alternative M2 macrophages. The M1 macrophage is involved in the inflammatory response, pathogen clearance, and anti-tumour immunity while the M2 phenotype mediates an anti-inflammatory response and wound healing, and has pro-tumour properties [44]. TAMs display an elevated expression of typical M2 phenotype markers such as CD163 and CD209 [14,21], indicating that TAMs closely resemble M2-polarized macrophages, which exhibit protumoural functions [20]. TAMs have been shown to promote tumour invasion, growth, angiogenesis, metastasis, and immunosuppression by releasing pro-tumour mediators such as MMPs, VEGF, and various chemokines such as CCL5 (RANTES) [15]. For example, TAM-derived CCL5 was reported to promote macrophages’ capacity for tumour repair and renewal in human epithelial ovarian cancer [45]. Thus, strategies to inhibit the recruitment of macrophages to tumour sites, the polarization of TAMs, and the release of pro-tumour mediators are considered promising targets for cancer therapy [46]. Recently, several natural compounds have been reported to have inhibitory effects on TAMs [47,48]. In this study, we have demonstrated that deoxyschizandrin inhibits M2 polarization and production of protumoural factors MMP-9, RANTES, and VEGF in macrophages stimulated by ovarian cancer cells. Furthermore, this suggested deoxyschizandrin may decrease metastasis and tumour repair in ovarian cancer tumour microenvironment.

In conclusion, our findings suggest that deoxyschizandrin induces growth arrest in human ovarian cancer cells and inhibits protumoural activities of TAMs (Figure S2). These findings expand our understanding of the health benefits and possible nutritherapeutic application of deoxyschizandrin and Schisandra berries.

## Figures and Tables

**Table 1:** Cytotoxic activity of deoxyschizandrin and schizandrin isolated from the berries of *S. chinensis* in human ovarian cancer cell lines.

	^a^ IC_50_		
	A2780	OVCAR3	SKOV3
Deoxyschizandrin (µM)	27.81 ± 3.44	70.34 ± 0.45	67.99 ± 5.91
Schizandrin (µM)	>100	>100	>100

Notes: ^a^ IC_50_ is defined as the concentration that results in a 50% decrease in the number of cells compared to that of the control groups. Human ovarian cancer cells were treated with various concentration of deoxyschizandrin for 48 h and cell viability was determined using MTT assay. The values represent the mean ± SD of the results from three independent experiments.

## Supplementary Material

The following are available online at www.mdpi.com/2072-6643/10/1/91/s1.
