# Supplementary material for: Deoxyschizandrin, Isolated from Schisandra Berries, Induces Cell Cycle Arrest in Ovarian Cancer Cells and Inhibits the Protumoural Activation of Tumour-Associated Macrophages
Source: Nutrients. 2018 Jan 15;10(1):91. doi: 10.3390/nu10010091 (PMC5793319; doi:10.3390/nu10010091)
Supplement: Supplementary file 1 [file nutrients-10-00091-s001.pdf]

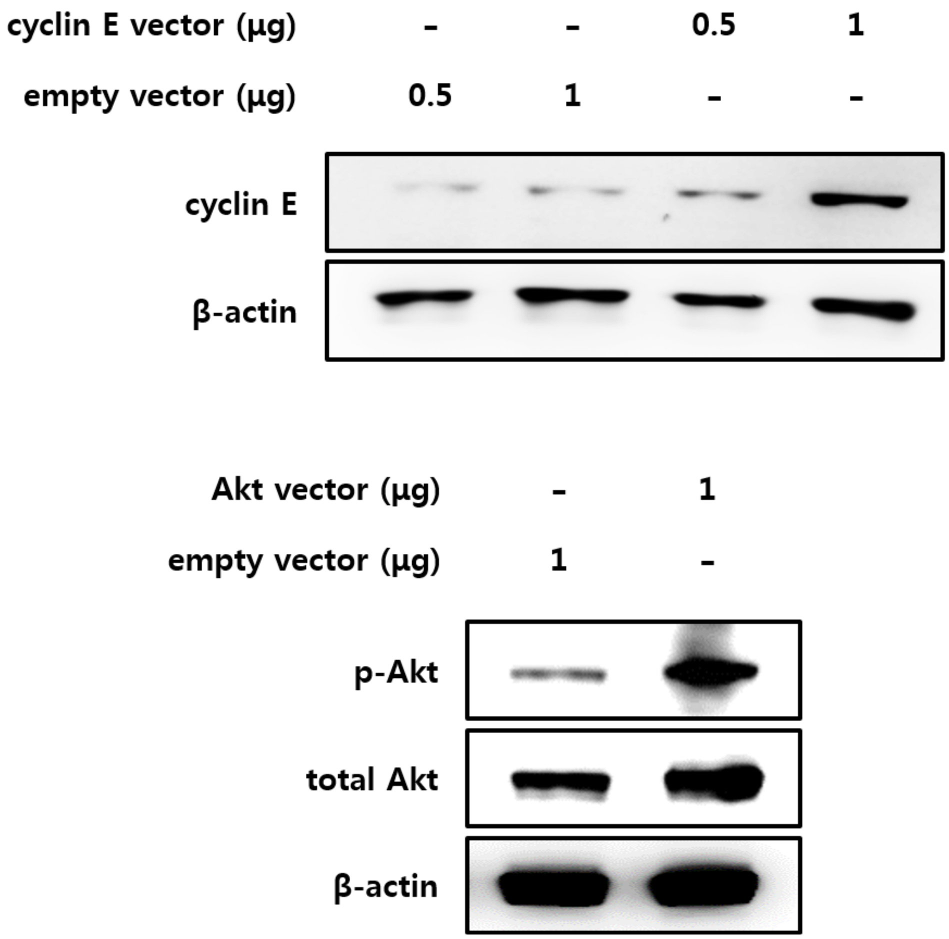

Supplementary Figure S1. A2780 cells were transfected with constitutively active cyclin E, Akt, and empty vector. The levels of proteins were measured using western blotting. β-actin was used as an internal control.

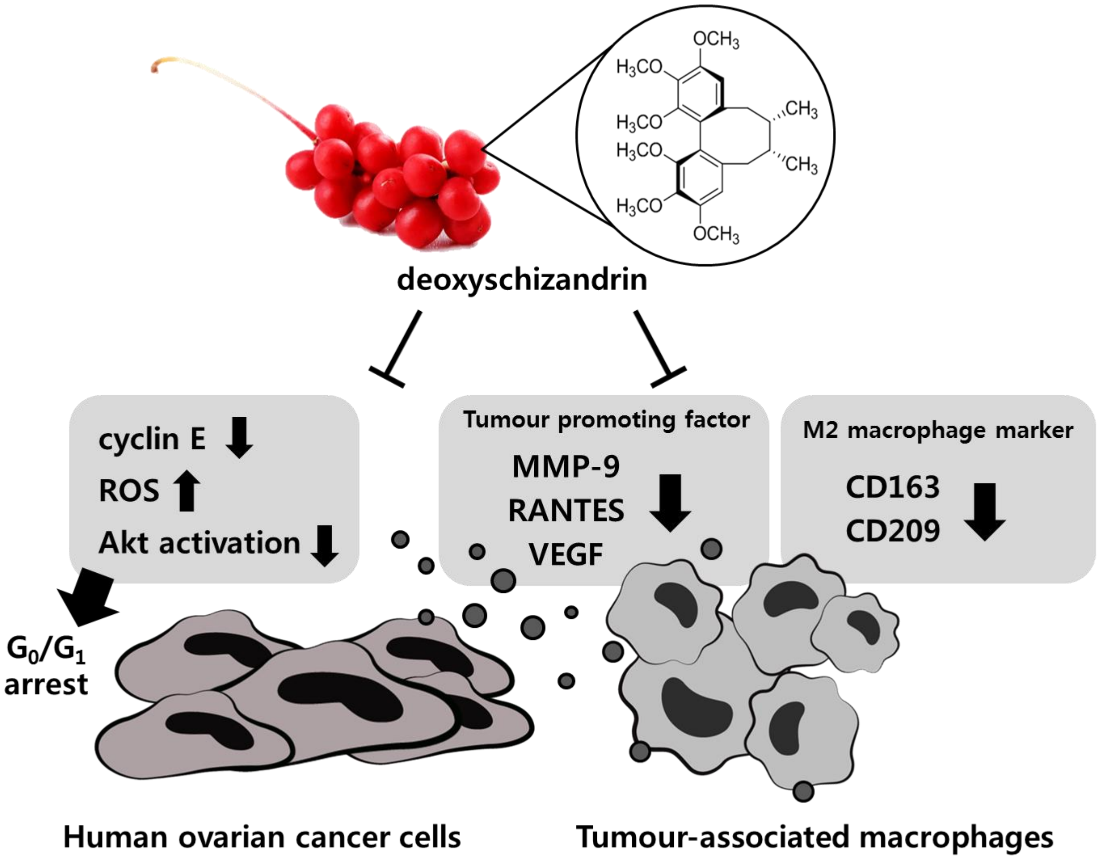

Supplementary Figure S2. Schematic of summary for the deoxyschizandrin-induced anti-tumour properties in human ovarian cancer cells.
